# New insights on microscopic properties of metal-porphyrin complexes attached to quartz crystal sensor

**DOI:** 10.1038/s41598-021-87773-z

**Published:** 2021-04-15

**Authors:** Haifa Alyousef, Badriah M. Alotaibi, Mohamed Ben Yahia, Meznah M. Alanazi, Norah A. Alsaif

**Affiliations:** 1 Department of Physics, College of Science, Princess Nourah Bint Abdulrahman University, Riyadh, Saudi Arabia; 2 Laboratory of Quantum and Statistical Physics LR18ES18, Faculty of Sciences of Monastir, 5000 Monastir, Tunisia

**Keywords:** Biochemistry, Chemical biology, Chemistry, Engineering, Materials science, Physics

## Abstract

A quartz crystal adsorbent coated with 5,10,15,20-tetrakis(4-methylphenyl) porphyrin was used to examine the complexation phenomenon of three metallic ions [aluminum(III), iron(III) and indium(III)]. The aim is to select the appropriate adsorbate for metalloporphyrin fabrication. The equilibrium adsorption isotherms of tetrakis(4-methylphenyl) porphyrin were performed at four temperatures (from 300 to 330 K) through the quartz crystal microbalance (QCM) method. Subsequently, the experimental data were analyzed in order to develop a thorough explanation of the complexation mechanisms. The experimental results indicated that the aluminum(III) chloride is the adequate material for metalloporphyrin application. Theoretical investigation was established through physics adsorption models in order to analyze the experimental isotherms. The AlCl_3_ isotherms were modeled via a single-layer adsorption model which is developed using the ideal gas law. Whereas, the FeCl_3_ isotherms were interpreted via a single-layer adsorption which includes the lateral interactions parameters (real gas law), indicating the lowest stability of the formed iron-porphyrin complex. The participation of the chloride ions in the double-layers adsorption of InCl_3_ was interpreted via layer by layer formulation. Interestingly, the physicochemical investigation of the three adopted models indicated that the tetrakis(4-methylphenyl) porphyrin adsorption was an endothermic process and that the aluminum(III) chloride can be recommended for an industrial application because it presents the highest adsorption energy (chemical bonds with porphyrins).

## Introduction

The investigation of the interaction between the porphyrin and the metals is the goal of this paper^1–3^. Thus, the metal-porphyrin complexes constitute the basic skeletons of the hemoglobin in the red blood cells^4^. They are also the main constituent of the chlorophyll which is the pigment of life responsible of the photosynthesis mechanism^5^. Moreover, the photosensitizing properties of the metal-porphyrin complexes have promoted to their use in the photodynamic therapy^6^. In addition, several reports^7–13^ demonstrated that these complexes could be used as potential ionophores of fluoride, chloride, nitrite…^10,12^. Therefore, the microscopic properties and the enzymatic activities of the metalloporphyrins complexes need to be extensively studied.

In recent papers, we found that the complexation of porphyrins with some charged metallic ions (zinc, platinum, magnesium…) has been investigated^14^. But, the use of porphyrins as complexing compounds of other metals such as the aluminium(III), the iron(III), and the indium(III) has not been totally studied and understood because the fabrication of these metalloporphyrins complexes is difficult, which prevented the progress of their application^15,16^.

**Figure 1:**
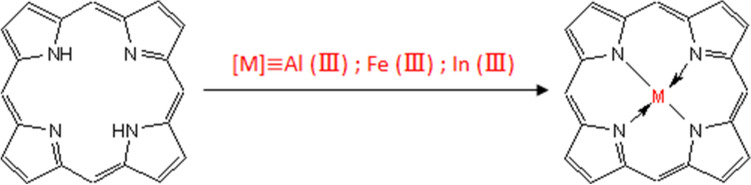
Illustration of the complexation reaction of the 5,10,15,20-tetrakis(4-methylphenyl) porphyrin with the metallic ions [aluminium(III)/iron(III)/indium(III)].

Interestingly, the first goal of this research paper is to control the interaction between these three ions [the aluminium(III) (Al^3+^), the iron (Fe^3+^) and the indium (In^3+^)] and the tetrakis(4-methylphenyl) porphyrin (Fig. 1) experimentally using the QCM method^17^. Indeed, thin layer of porphyrins was doped onto the quartz crystal surface by the spin coating method^18^. The design of chemically modified electrodes, where stable complexes of porphyrins can be formed with the ions (Al^3+^, Fe^3+^ and In^3+^), allows the determination of the adsorption isotherms which characterize the proper metal once it has been inserted into the porphyrin ring^19^.

The second goal of this paper is devoted to the investigation of the microscopic properties of the formed complexes using an advanced theoretical modeling of the experimental data^20,21^. Hence, the advanced modeling analysis is developed based on the statistical physics adsorption models^20–22^ which are used to describe the new vision of the experimental adsorption isotherms of the three complexation systems.

Actually, there are some models in literature that can describe the profile of the adsorption isotherms such us the Langmuir and the BET models^19,23^. These models provide an interpretation of the adsorption isotherms with empirical aspect. In fact, they provide an estimation of the monolayer value of the adsorbed quantity on the surface and the adsorption energy. However, they do not provide any physical indication about the adsorption mechanism. Overall, from the statistical physics models, the physical parameters values calculated from numerical simulation^21^ are used to outline the adsorption process at the ionic scale if the adsorbate and the adsorbent properties are well known. However, to date, no one equation gives accurate results throughout the whole range of the adsorbate concentrations, and for all types of particles. The novelty of our paper and the progress against recent works is to develop advanced models expressions which provide physicochemical properties of the adsorption process of the three studied ions on porphyrins at the microscopic level. The previous metal-porphyrin systems were studied by the empirical models or by the statistical physics models established on the basis of the ideal gas approach in which the mutual interactions between the particles are neglected^22^. In this paper, in addition to the classical models, advanced forms of the single-layer, the double-layers and the multi-layers models that take into account the lateral interactions between the particles are developed and adopted for the description of the adsorption systems. They are established based on the use of the chemical potential of the real gas and they have not been published previously in any of previous papers.

Then, the fundamental aim of the modeling work is to find the sufficient systematic model that can anticipate physical characteristics of porphyrins adsorption isotherms based on the physicochemical parameters of the adopted model. The experimental and the theoretical results will be investigated by an energetic analysis of the complexation energies of the three metal ions in order to evaluate the stability of the three formed complexes^20–22^.

## Experimental measurements of adsorption isotherms

### Experimental QCM setup

**Figure 2:**
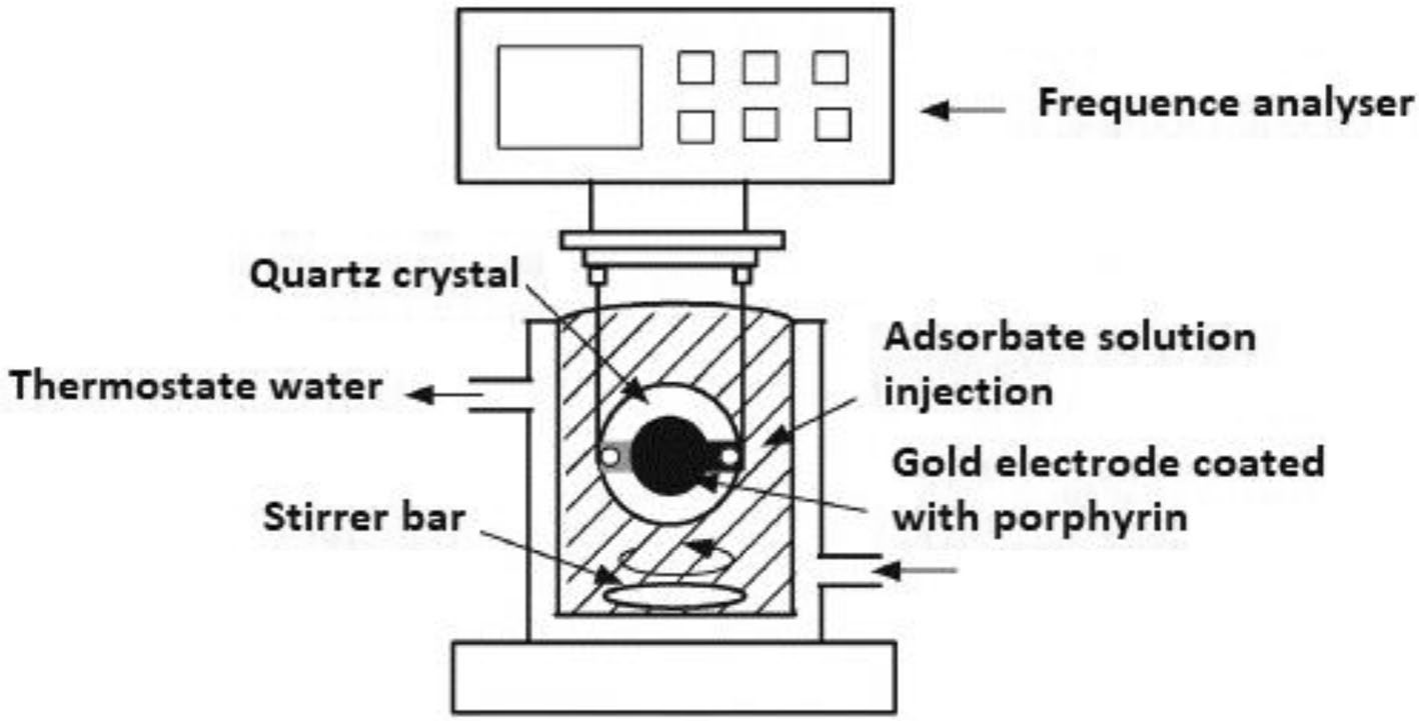
Experimental setup of quartz crystal microbalance strategy devoted for the achievement of experimental adsorption isotherms of AlCl_3_, FeCl_3_ and InCl_3_ on the 5,10,15,20-tetrakis(4-methylphenyl) porphyrin.

The experimental QCM setup is presented in Fig. 2.

The QCM measurements were performed based on the piezoelectric quartz crystal (polished crystal which has a thickness of about 331 μm and a diameter of 2.54 cm)^24,25^.

The quartz crystal, which is covered with a layer of gold on both sides, is brought into resonance by means of an alternating electric current. For the adsorption measurement, the adsorbent was doped onto the clean crystal surface by spin coating technique. In the bain-marie (reactor with 100 mL of pure water), the adsorption cell was placed in a Teflon probe and it was covered by a ring in order to protect the electrode from the penetration of liquid. The connection of the probe with the frequency-counter monitor was assured by means of a coaxial cable. Then, 15 injections of adsorbate (AlCl_3_/FeCl_3_/InCl_3_) were added in the reactor in order to increase the concentration of the metallic ions. The frequency-counter monitor indicated the frequency variation corresponding to the final concentration after each adsorbate addition.

**Figure 3:**
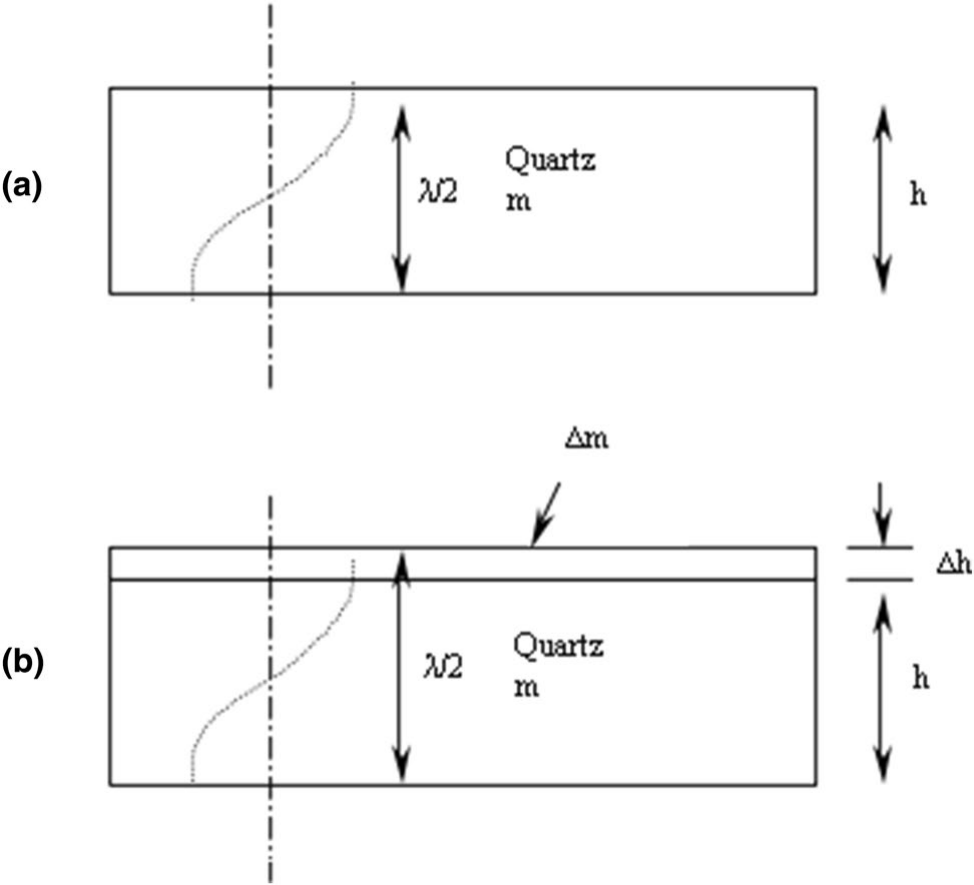
Sauerbrey's model of an oscillating quartz crystal: (**a**) before mass addition and (**b**) after mass addition.

A slight frequency variation was observed after each adsorbate solution addition (slight mass variation of one of the crystal electrodes). This effect was modeled by Sauerbrey in 1959^26^ (Fig. 3).

According to Fig. 3, oscillating quartz with a thickness h and without deposited mass m gives the following resonance frequency (*f*_*0*_):

1 $$ f_{0} = \upsilon /\lambda = \upsilon /2h $$

where, λ (λ = υ * *f*_*0*_) is the propagation of acoustic wavelength which is twice the thickness of the quartz (The resonance condition verifying that a half wavelength is confined in the thickness of the resonator) and υ is the propagation speed of the acoustic wave (3336 × 10^5^ cm/s).

Then, the addition of mass to the quartz surface creates an increase in the thickness (∆*h*) which causes a resonant frequency change (∆*f*). Therefore, the rise of the thickness (mass) induces a decrease in the frequency in accordance with the following equations:

2 $$ \frac{\Delta f}{{f_{0} }} = - \frac{\Delta h}{h} $$

3 $$ \frac{\Delta f}{{f_{0} }} = - \frac{\Delta m}{m} $$

where, the mass variation (∆*m*) can be written as a function of the crystal density *ρ* (g/cm^3^) and the sensitive surface of quartz *A* (cm^2^).

4 $$ \Delta m = \rho \cdot \Delta h \cdot A $$

By combining these equations, we obtain the following expression^27^:

5 $$ \Delta f = - \left( {\frac{{2 \cdot f_{0}^{2} }}{A \cdot \rho \cdot \upsilon }} \right) \cdot \Delta m $$

This equation is called the Sauerbrey’s equation which can be otherwise written^26,28^:

6 $$ \Delta m = - \frac{\Delta f}{C} $$

where, C is the linear sensitivity factor (Hz cm^2^/μg) which is the specific characteristic of the crystal.

Finally, we apply the Sauerbrey's hypothesis to compute the deposited masses *A*_*Q*_ of In^3+^, Al^3+^ and Fe^3+^ on the 5,10,15,20-tetrakis(4-methylphenyl) porphyrin.

### Experimental data discussion

**Figure 4:**
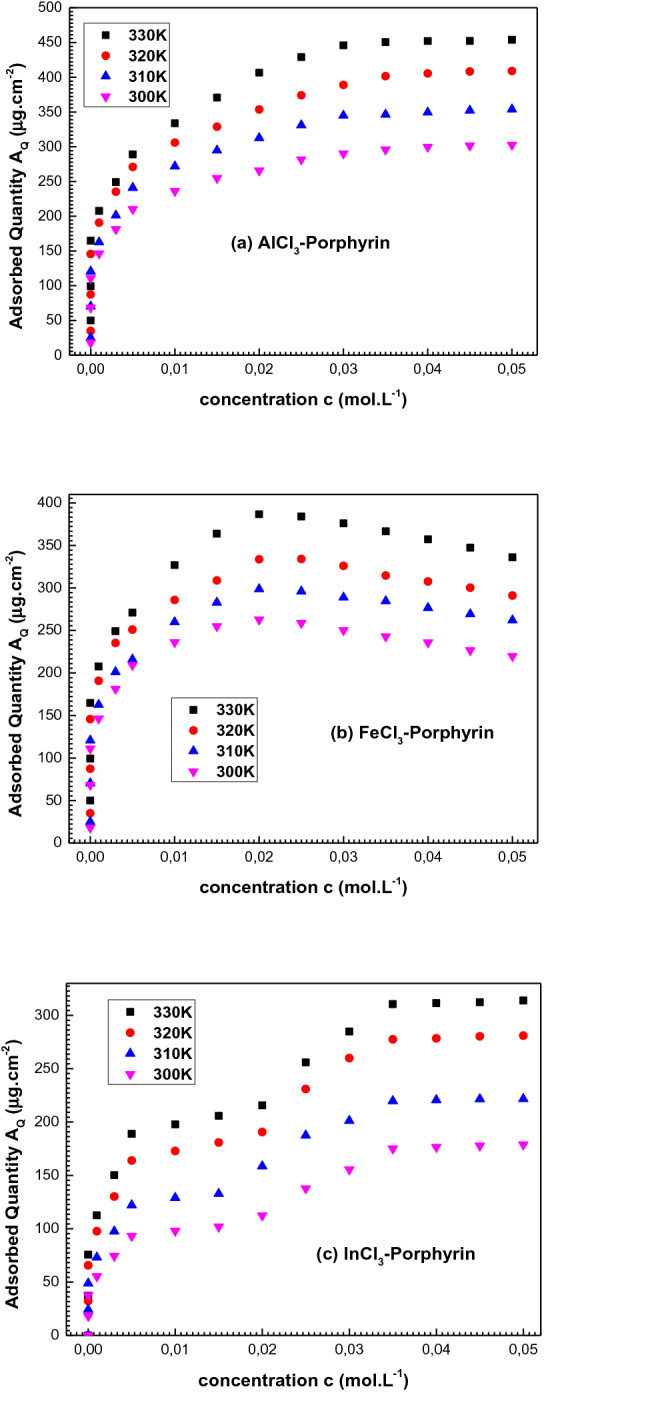
Equilibrium adsorption isotherms of AlCl_3_, FeCl_3_ and InCl_3_ onto 5,10,15,20-tetrakis (4-methylphenyl) porphyrin (H_2_TTPP) measured at four adsorption temperatures (300–330 K).

Firstly, the adsorbed amounts depicted with the adsorption isotherms (Fig. 4) confirm that the complexation of the tetrakis(4-methylphenyl) porphyrin by the three metallic ions In^3+^, Al^3+^ and Fe^3+^ was carried out at all the temperatures. It is confirmed that the tetrakis(4-methylphenyl) porphyrin should be a chemical sensor of the three metals.

Secondly, by comparing the performance of the three adsorption systems in terms of quantity, we can note the following order of the adsorption performance: A_Q_ (AlCl_3_) > A_Q_ (FeCl_3_) > A_Q_ (InCl_3_). The adsorbed quantities are the highest for AlCl_3_. Then, the aluminum chloride is the best adsorbate compound for the tetrakis(4-methylphenyl) porphyrin complexation.

Lastly, it is clear from the experimental data of the three adsorption systems that the AlCl_3_ isotherms show a unique stable saturation level for all the temperatures, the adsorbed quantities of FeCl_3_ decrease after the saturation level and the InCl_3_ isotherms present two stability states. It should be suggested that the tested porphyrin adsorb only one layer of cationic metal for AlCl_3_ and FeCl_3_ while, many adsorbed layers are formed in the case of InCl_3_. The multi-layers ionic adsorption of the indium chloride takes place via the layer by layer (LBL) process which is based on charge neutralization between particles having opposite charge signs (anions and cations)^20,29^.

In the following section, the microscopic investigation of these experimental observations is carried out through the physical modeling of the experimental data.

## Theoretical modeling of adsorption isotherms by statistical physics treatment

### Adsorption models development

According to the adsorption isotherms of tetrakis(4-methylphenyl) porphyrin (Fig. 4), we can notice two phenomena: single-layer adsorption of AlCl_3_ and FeCl_3_, and LBL multi-layers adsorption of InCl_3_. The experimental isotherms can be analyzed via an analytical physical modeling in the light of the statistical physics treatment.

The first progress of this advanced treatment is seen against the oldest empirical equation elaborated by Langmuir et al.^19^. The Langmuir model expects that an adsorbent site can utmost integrate one particle however our statistical physics models guess that one receptor site can suit n particles where n is a variable number. In addition, our statistical physics models assume the presence of various adsorption energies for various receptor sites, while the empirical models just accept the presence of one adsorption energy level for all the adsorbent sites. Furthermore, the statistical physics models give information about the number of adsorbed layers during the adsorption mechanism whereas, the Langmuir model assumes that one adsorbed layer is formed during the adsorption process. It should be also mentioned that in the case of the multi-layers ionic adsorption, we have fundamentally to use a model that reflects a layer by layer adsorption^20^. The empirical models do not assess this supposition.

In reality, the analytical development of the statistical physics models requires to take account of some assumptions:

First of all, it is assumed that the adsorption system can be studied through the grand-canonical ensemble of Gibbs demonstrated in previous works^30,31^. Thus, the complexation reaction involving the free phase (AlCl_3_/FeCl_3_/InCl_3_) and the tested adsorbent (tetrakis(4-methylphenyl) porphyrin) is summarized in Eq. (7^30,32^:

7 $$ {\text{n}}({\text{I}}) + {\text{P}} \rightleftarrows ({\text{I}})_{{\text{n}}} - {\text{P}} $$

where, I is the adsorbate ion in liquid phase, P is the 5,10,15,20-tetrakis(4-methylphenyl) porphyrin molecules in solid state, (I)_n_–P is the Ion(III)–Porphyrin complex and n is the stoichiometric coefficient of the adsorption reaction. It represents the number of bonded ions per adsorbent site. In general, this parameter can identify the nature of the adsorption process (n ≤ 0.5: multi-interaction process, n ≥ 1 multi-ionic process).

The studied system, which is supposed in the grand-canonical situation, is characterized by the chemical potential (μ) and the temperature (T) imposing from the outside towards the considered system. These variables are included in the general expression of the partition-function of the grand-canonical ensemble (z_gc_) which is the starting point for each model development^30–32^.

Concerning the adsorption via a formation of one adsorbed layer, we take account of one energy level (− E). For the double-layers and the multi-layers adsorption processes (LBL adsorptions), two energies (− E_1_) and (− E_2_) can be responsible for this process. Note that the first energy (− E_1_) characterizes the adsorption of the first layer; and the second energy (− E_2_) is in relationship with the formation of the additional formed layers^29,32^.

The next stage of this physical modeling consists of calculating the average number (N_0_) of identical occupied porphyrins sites (P_m_) which has the following expression^30^:

8 $$ N_{0} = Nk_{B} T\frac{{\partial \ln (z_{gc} )}}{\partial \mu } $$

Here, we apply the chemical potential coupled to the ideal gas approach (μ_p_). In the presence of this mean potential, we can consider that one individual particle has no interaction with the rest of the system like an ideal fermions gas of electrons. It can be written as a function of the partition-function of translation (z_Tr_) and the number of adsorbates (N)^29–31^:

9 $$ \mu_{p} = \frac{1}{\beta }\ln \left( {\frac{N}{{z_{Tr} }}} \right) $$

The same modeling work is also performed using the chemical potential of a real gas (µ_r_). In this case, the lateral interactions between the adsorbates at free state are taken into account and the cohesion pressure a and the covolume b are included in the expression of µ_r_^20,33^:

10 $$ \mu_{r} = \mu_{p} + k_{B} T\ln \frac{1}{1 - bc} + k_{B} T\frac{bc}{{1 - bc}} - 2ac $$

Finally, the adsorbed amount expression (*A*_*Q*_) of each physical model is determined by the next equation^30,31,33^:

11 $$ A_{Q} = n \times N_{0} $$

Table 1 summarizes the development of the six physical models expressions.

**Table 1:** Analytical expressions of the grand-canonical partition function (z_gc_) and the adsorbed quantity (A_Q_) corresponding to the single-layer models, the double-layers models and the multi-layers models.

Adsorption model	Ideal gas approach [µ_p_, Eq. (9)]	Real gas approach [µ_r_, Eq. (10)]
Single-layer model	$z_{gc} = 1 + e^{{\beta (E + \mu_{p} )}}$ $A_{Q} = \frac{{nP_{m} }}{{1 + \left( {\frac{{c_{1/2} }}{c}} \right)^{n} }}$ _Where c1/2 is:_ $c_{1/2} = Se^{{ - \frac{{E_{1/2} }}{{k_{B} T}}}}$	$z_{gc} = 1 + e^{{\beta (E + \mu_{r} )}}$ $A_{Q} = \frac{{nP_{m} }}{{1 + \left( {w_{1/2} \frac{1 - bc}{c}e^{2\beta ac} e^{{ - \frac{bc}{{1 - bc}}}} } \right)^{n} }}$ _Where w1/2 is:_ $w_{1/2} = Se^{{ - \frac{{E_{1/2} }}{{k_{B} T}}}}$
Double-layers model	$z_{gc} = 1 + e^{{\beta (E_{1} + \mu_{p} )}} + e^{{\beta (E_{1} + E_{2} + 2\mu_{p} )}}$ $A_{Q} = nP_{m} \frac{{\left( {\frac{c}{{c_{1} }}} \right)^{n} + 2\left( {\frac{c}{{c_{2} }}} \right)^{2n} }}{{1 + \left( {\frac{c}{{c_{1} }}} \right)^{n} + \left( {\frac{c}{{c_{2} }}} \right)^{2n} }}$ _Where c1 and c2 are:_ $c_{{1,2}} = Se^{\frac{{E_{{1,2}} }}{{k_{B} T}}}$	$z_{gc} = 1 + e^{{\beta (E_{1} + \mu_{r} )}} + e^{{\beta (E_{1} + E_{2} + 2\mu_{r} )}}$ $A_{Q} = nP_{m} \frac{{\left( {\frac{c}{{w_{1} (1 - bc)e^{2\beta ac} e^{{ - \frac{bc}{{1 - bc}}}} }}} \right)^{n} + 2\left( {\frac{c}{{w_{2} (1 - bc)e^{2\beta ac} e^{{ - \frac{bc}{{1 - bc}}}} }}} \right)^{2n} }}{{1 + \left( {\frac{c}{{w_{1} (1 - bc)e^{2\beta ac} e^{{ - \frac{bc}{{1 - bc}}}} }}} \right)^{n} + \left( {\frac{c}{{w_{2} (1 - bc)e^{2\beta ac} e^{{ - \frac{bc}{{1 - bc}}}} }}} \right)^{2n} }}$_Where w1 and w2 are:_ $w_{{1,2}} = Se^{{ - {\text{ }}\frac{{E_{{1,2}} }}{{k_{B} T}}}}$
Multi-layers model	$$\begin{gathered} z_{gc} = 1 + e^{{\beta (E_{1} + \mu_{p} )}} \hfill \\ + \sum\limits_{{N_{i} = 2}}^{L} {e^{{ - \beta \left( { - E_{1} - (N_{i} - 1)E_{2} - N_{i} \mu_{p} } \right)}} } \hfill \\ \end{gathered}$$ $A_{Q} = nP_{m} \times \left( {\frac{{\left( {\frac{c}{{c_{1} }}} \right)^{n} + \left( {\frac{c}{{c_{1} }}} \right)^{n} \left( {\frac{c}{{c_{2} }}} \right)^{n} \left( {1 - 2\left( {\frac{c}{{c_{2} }}} \right)^{nL} - L\left( {\frac{c}{{c_{2} }}} \right)^{n(L + 1)} + \frac{{\left( {\frac{c}{{c_{2} }}} \right)^{n} \left( {1 - \left( {\frac{c}{{c_{2} }}} \right)^{nL} } \right)}}{{1 - \left( {\frac{c}{{c_{2} }}} \right)^{n} }}} \right)}}{{\left( {1 - \left( {\frac{c}{{c_{1} }}} \right)^{n} } \right)\left( {1 - \left( {\frac{c}{{c_{2} }}} \right)^{n} } \right) + \left( {\frac{c}{{c_{1} }}} \right)^{n} \left( {\frac{c}{{c_{2} }}} \right)^{n} \left( {1 - \left( {\frac{c}{{c_{2} }}} \right)^{nL} } \right)}}} \right)$_Where c1 and c2 are:_ $c_{{1,2}} = Se^{\frac{{E_{{1,2}} }}{{k_{B} T}}}$	$$\begin{gathered} z_{gc} = 1 + e^{{\beta (E_{1} + \mu_{r} )}} \hfill \\ + \sum\limits_{{N_{i} = 2}}^{L} {e^{{ - \beta \left( { - E_{1} - (N_{i} - 1)E_{2} - N_{i} \mu_{r} } \right)}} } \hfill \\ \end{gathered}$$ $A_{Q} = nP_{m} \times \left( {\frac{{\left( {\frac{c}{{c_{1} }}} \right)^{n} + \left( {\frac{c}{{c_{1} }}} \right)^{n} \left( {\frac{c}{{c_{2} }}} \right)^{n} \left( {1 - 2\left( {\frac{c}{{c_{2} }}} \right)^{nL} - L\left( {\frac{c}{{c_{2} }}} \right)^{n(L + 1)} + \frac{{\left( {\frac{c}{{c_{2} }}} \right)^{n} \left( {1 - \left( {\frac{c}{{c_{2} }}} \right)^{nL} } \right)}}{{1 - \left( {\frac{c}{{c_{2} }}} \right)^{n} }}} \right)}}{{\left( {1 - \left( {\frac{c}{{c_{1} }}} \right)^{n} } \right)\left( {1 - \left( {\frac{c}{{c_{2} }}} \right)^{n} } \right) + \left( {\frac{c}{{c_{1} }}} \right)^{n} \left( {\frac{c}{{c_{2} }}} \right)^{n} \left( {1 - \left( {\frac{c}{{c_{2} }}} \right)^{nL} } \right)}}} \right)$_Where c1 and c2 are functions of w1 and w2:_ $c_{1,2} = w_{1,2} (1 - bc)e^{2\beta ac} e^{{ - \frac{bc}{{1 - bc}}}}$ and $w_{{1,2}} = Se^{{ - {\text{ }}\frac{{E_{{1,2}} }}{{k_{B} T}}}}$

All models expressions are developed through the statistical physics formalism and constitute advanced forms of the empirical expression of the Langmuir model. The difference between the classical models (ideal gas approach) and the advanced forms(real gas approach) is essentially seen in the number of parameters in the analytical expressions of the models but they contain in their expressions parameters that are important from a physical point of view, unlike empirical forms, whose parameters generally have no physical meaning.

### Fitting of adsorption isotherms with the analytical models

The six adsorption models (Table 1) were applied on all experimental isotherms by the intermediate of a numerical fitting program^29^. The criterions to select the best model are the RMSE coefficient (Residual-root-mean-square-error), the AIC coefficient (Akaike-information-criterion) and the determination coefficient R^2^.

Table 2 shows the values of the three adjustment coefficients.

**Table 2:** Values of the correlation coefficient R^2^, the residual root mean square coefficient RMSE and the Akaike information criterion AIC deduced from the numerical adjustment of experimental isotherms with the three statistical physics models.

Statistical physics model		Single-layer model (ideal gas)			Single-layer model (real gas)			Double-layers model (ideal gas)		
Adjustment coefficient		R^2^	RMSE	AIC	R^2^	RMSE	AIC	R^2^	RMSE	AIC
AlCl_3_-porphyrin	300 K	*0.99*	*1.22*	*15.02*	0.94	2.88	19.84	0.91	2.99	23.64
	310 K	*0.98*	*1.56*	*18.71*	0.95	2.97	20.34	0.92	2.93	23.84
	320 K	*0.99*	*1.34*	*17.64*	0.93	2.56	19.65	0.89	3.01	21.98
	330 K	*0.98*	*1.89*	*16.76*	0.94	3.24	20.89	0.91	4.02	22.76
FeCl_3_-porphyrin	300 K	0.84	5.12	28.91	*0.98*	*1.11*	*20.3*	0.91	2.98	24.61
	310 K	0.82	5.49	28.23	*0.99*	*1.23*	*21.44*	0.93	3.07	25.94
	320 K	0.82	4.99	28.99	*0.97*	*1.32*	*20.98*	0.91	3.51	25.16
	330 K	0.82	5.01	28.54	*0.97*	*1.29*	*21.52*	0.92	3.11	25.28
InCl_3_-porphyrin	300 K	0.71	7.92	36.7	0.88	5.64	30.72	*0.99*	*2.11*	*27.64*
	310 K	0.65	8.13	39.4	0.87	6.78	32.65	*0.99*	*1.99*	*26.91*
	320 K	0.64	8.13	38.8	0.82	6.36	31.47	*0.97*	*1.98*	*26.98*
	330 K	0.67	7.62	38.4	0.79	5.73	32.28	*0.97*	*2.02*	*26.56*

The italic values are the values of adjustement coefficients (R^2^/RMSE/AIC) of the best model devoted for the microscopic description of each experimental adsorption system (AlCl_3_-porphyrin/FeCl3-porphyrin/InCl3-porphyrin)

According to Table 2, the experimental data of AlCl_3_ can be interpreted by the single-layer model (ideal gas approach) whereas; the adsorption isotherms of FeCl_3_ show the best coefficients of adjustment with the single-layer model of real gas. This explains that the decline of the FeCl_3_ isotherms at high equilibrium concentration is fundamentally due to the lateral interaction impacts and confirms that the aluminum ions are the best compounds for porphyrin complexation in terms of stability. On the other hand, the LBL double-layers model (ideal gas approach) is selected for the theoretical description of the InCl_3_ adsorption. In this case, two adsorbed layers are formed based on charge neutralization between cations (In^3+^) and anions (Cl^−^).

Overall, the numerical fitting results shows that AlCl_3_ is the appropriate adsorbate for the tetrakis(4-methylphenyl) porphyrin complexation considering that the complexation process is carried out without lateral interactions influences compared to the FeCl_3_ adsorption and considering that the chloride particles (Cl^−^) are remained in solution and do not have an effect on the complexation process compared to the InCl_3_ adsorption.

It should be noted that the adsorption models (Table 1) presents some physicochemical parameters which should be used for the microscopic interpretation of the three complexation mechanisms: for AlCl_3_, the single-layer model (ideal gas) includes three parameters (the number of aluminum per porphyrin site n, the density of porphyrin sites P_m_ and an energetic parameter c_1/2_). For FeCl_3_, the single-layer model (real gas) presents the parameter a (cohesion pressure) and the parameter b (covolume) in addition to the steric parameters n and P_m_ and the energetic parameter w_1/2_. The LBL adsorption of InCl_3_ can be interpreted via four physicochemical variables [n and P_M_ (steric variables), and c_1_ and c_2_ (energetic variables)].

In the next section, the fitting values of these variables are analyzed and discussed versus the temperature in order to investigate the three complexation processes at the ionic level.

## Physicochemical interpretation of the three complexation processes

We give in Table 3 all the fitting values of the steric and the energetic variables affecting the reaction of AlCl_3_, FeCl_3_ and InCl_3_ with the tetrakis(4-methylphenyl) porphyrin at the four temperatures.

**Table 3:** Fitting values of the steric parameters (n and P_m_), the Van der Waals parameters (a and b) and the energetic parameters (c_1/2_, w_1/2_, c_1_ and c_2_) affecting the adsorption of AlCl_3_, FeCl_3_ and InCl_3_ on 5,10,15,20-tetrakis(4-methylphenyl) porphyrin at four temperatures.

Adsorption isotherm/fitting model	Models’ parameters	300 K	310 K	320 K	330 K
AlCl_3_/single-layer model (ideal gas)	n	0.81	0.89	0.99	1.01
	P_m_	320.6	366.1	401.9	450.7
	c_1/2_	0.015	0.016	0.016	0.017
FeCl_3_/single-layer model (real gas)	n	0.73	0.79	0.84	0.89
	P_m_	262.6	298.7	333.6	389.8
	w_1/2_	0.011	0.012	0.011	0.012
	a	7.2 × 10^–9^	6.1 × 10^–9^	5.8 × 10^–9^	5.1 × 10^–9^
	b	3.2 × 10^–12^	3.9 × 10^–12^	4.8 × 10^–12^	5.7 × 10^–12^
InCl_3_/Double-layers model (ideal gas)	n	0.66	0.69	0.74	0.82
	P_m_	189.4	233.8	289.4	320.5
	c_1_	0.003	0.0035	0.0032	0.0036
	c_2_	0.025	0.026	0.025	0.027

### Steric study and lateral interactions influence

The parameters n and P_M_ are typified by a steric aspect. The product of these parameters is the result of the maximum adsorption capacity^34^.

The fitted values of n give information about the number of metallic ions that can be interact with one adsorbent site. Based on Table 3, all n values of the tetrakis(4-methylphenyl) porphyrin adsorption are found inferior to 1 for the three complexation systems. Therefore, it can be concluded that the AlCl_3_, the FeCl_3_ and the InCl_3_ adsorptions were only governed by a multi-interaction mechanism at all the temperatures^35^.

The fitted values of P_m_ describe the number of porphyrins sites accessible to ions at each temperature. Table 3 demonstrates that the adsorptions of AlCl_3_ and FeCl_3_ present the highest values of P_m_ at all the temperatures: P_m_ (AlCl_3_) > P_m_ (FeCl_3_) > P_m_ (InCl_3_). In fact, the anions are not involved in the complexation processes of AlCl_3_ and FeCl_3_ so there is a fast insertion of the metallic ions in the porphyrin cavities. However, the contribution of the anionic particles in the InCl_3_ adsorption prevents the complexation of porphyrin by the indium ions because of the interaction between the two adsorbed layers.

Furthermore, despite the AlCl_3_ and the FeCl_3_ adsorptions are both single-layer adsorption processes, where there is no contribution of the chloride ions at the layer formation, the fitted values of P_m_ are the lowest for FeCl_3_. Thus, the physical model that describes the FeCl_3_ adsorption includes the lateral interactions effect by the intermediate of the parameter a (cohesion pressure) and the parameter b (covolume)^20^. It can be concluded that the decrease of FeCl_3_ isotherms (Fig. 4b) can be the result of the high adsorbate–adsorbate interaction which reflects a weak binding Fe^3+^-Porphyrin comparing to the Al^3+^-Porphyrin binding.

Overall, it can be concluded that the use of the aluminum chloride guarantees more stability during the metalloporphyrin formation.

### Energetic study

The molar adsorption energies should be calculated by means of the energetic coefficients which are deduced from fitting the experimental isotherms with the three physical models^36^.

The single-layer model (ideal gas) includes one energetic variable c_1/2_. The adsorption energy of AlCl_3_ can be determined via the following expression:

12 $$ - E_{1/2} = \frac{1}{\beta }*\ln \left( {{\raise0.7ex\hbox{${c_{1/2} }$} \!\mathord{\left/ {\vphantom {{c_{1/2} } {S(Al{\text{Cl}}_{{3}} )}}}\right.\kern-\nulldelimiterspace} \!\lower0.7ex\hbox{${S(Al{\text{Cl}}_{{3}} )}$}}} \right) $$

where, S(AlCl_3_) is the solubility of the aluminum(III) chloride in aqueous solution.

The single-layer model (real gas) gives rise to the parameter w_1/2_. The adsorption energy of FeCl_3_ is determined by the intermediate of the adjusted value of w_1/2_ through the following formula:

13 $$ - E_{1/2} = \frac{1}{\beta }*\ln \left( {{\raise0.7ex\hbox{${w_{1/2} }$} \!\mathord{\left/ {\vphantom {{w_{1/2} } {S(Fe{\text{Cl}}_{{3}} )}}}\right.\kern-\nulldelimiterspace} \!\lower0.7ex\hbox{${S(Fe{\text{Cl}}_{{3}} )}$}}} \right) $$

where, S(FeCl_3_) is the water solubility of iron(III) chloride.

For InCl_3_, the double-layers model introduces the variables c_1_ and c_2_ with energetic aspect:

14 $$ - E_{1} = \frac{1}{\beta }*\ln \left( {{\raise0.7ex\hbox{${c_{1} }$} \!\mathord{\left/ {\vphantom {{c_{1} } {S(In{\text{Cl}}_{{3}} )}}}\right.\kern-\nulldelimiterspace} \!\lower0.7ex\hbox{${S(In{\text{Cl}}_{{3}} )}$}}} \right) $$

15 $$ - E_{2} = \frac{1}{\beta }*\ln \left( {{\raise0.7ex\hbox{${c_{2} }$} \!\mathord{\left/ {\vphantom {{c_{2} } {S(In{\text{Cl}}_{{3}} )}}}\right.\kern-\nulldelimiterspace} \!\lower0.7ex\hbox{${S(In{\text{Cl}}_{{3}} )}$}}} \right) $$

where, S(InCl_3_) is the solubility of the indium(III) chloride in aqueous solution.

According to Table 4, for the indium(III) chloride adsorption, it is obviously remarked that the calculated values of |− E_1_| which characterizes the indium-porphyrin interaction are greater than those of |− E_2_| (interaction between the adsorbed layers^37^). Therefore, we can conclude that the interaction |− E_1_| should be compared to the others adsorption systems energies (|− ΔE_1/2_|) to evaluate the stability of the formed metalloporphyrin complexes. The adsorption energies |− E_1_| of InCl_3_ and |− E_1/2_| of AlCl_3_ and FeCl_3_ are the determining factors of the choice of the finest adsorbate because they characterizes the direct interaction between the three metallic ions and the tetrakis(4-methylphenyl) porphyrin.

**Table 4:** Values of adsorption energies |− E_1/2_| for AlCl_3_ and FeCl_3_ adsorptions and |− E_1_| and |− E_2_| for InCl_3_ adsorption given in modulus values at 300, 310, 320 and 330 K.

Adsorption temperature (K)		300 K	310 K	320 K	330 K
AlCl_3_-porphyrin	|− E_1/2_| (kJ/mol)	69.5	70.6	75.4	80.3
FeCl_3_-porphyrin	|− E_1/2_| (kJ/mol)	36.4	38.7	40.8	43.1
InCl_3_-porphyrin	|− E_1_| (kJ/mol)	30.4	33.5	36.8	38.7
	|− E_2_| (kJ/mol)	18.4	21.3	23.7	26.8

Based on Table 4, we observe that: |− E_1/2_| (AlCl_3_) > |− E_1/2_| (FeCl_3_) > |− E_1_| (InCl_3_). This result indicates that the affinity of the tetrakis(4-methylphenyl) porphyrins cavities to the aluminum ions is more important compared to FeCl_3_ and InCl_3_ which confirms that aluminum(III)-porphyrin is the best adsorption system suited for metalloporphyin complexes application.

Moreover, |− E_1/2_| (FeCl_3_) and |− E_1_| (InCl_3_) are below 40 kJ/mol^22^: the bindings iron(III)-porphyrin and indium(III)-porphyrin are carried out via physical process. Whereas, the adsorption energies |− E_1/2_| of AlCl_3_ are superior to 40 kJ/mol for all the temperatures. For this system, the adsorption is carried out via a chemical process involving covalent bonds^31^.

As a conclusion, the steric study and the energetic interpretation confirm the suggestion of the aluminum(III) for the metal-porphyrin complex facrication.

### Temperature influence on the complexation processes

**Figure 5:**
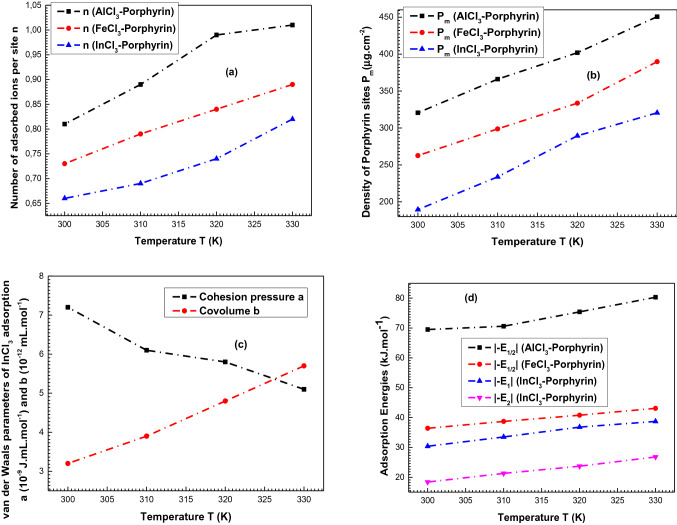
Variations of the adjusted values of the steric parameters (n and P_m_), the van der Waals parameters (**a**, **b**) and the adsorption energies (|− E_1/2_|, |− E_1_| and |(− E_2_)|) as a function of temperature (300–330 K).

It is noted from Fig. 4 that the temperature exerts exactly the same influence on the three complexation systems: once the temperature increases, the adsorption capacities increase. This can be explained from Fig. 5 which shows that the values of the coefficients n (Fig. 5a) and P_m_ (Fig. 5b) rise with the temperature from 300 to 330 K. It can be concluded that the thermal agitation effect favors the adsorption dynamics which was an endothermic process^38^: the rise of the temperature active other receptor sites to contribute in the complexation process.

From Fig. 5c, it is noted that the parameter a decreases with the expansion of the temperature for the FeCl_3_ adsorption while the covolume b increases. The decrease of the cohesion pressure indicates that the lateral interactions effect is low at high temperature. The increase of the parameter b reflects a strong distance between the adsorbates^20^. The behaviors of these two parameters explain the highest reproducibility of iron adsorption at 330 K and demonstrate the endothermic criterion of the studied process.

A last remark from Fig. 5d: it can be seen that all the adsorption energies rise with the expansion of the temperature from 300 to 330 K. This can be interpreted by the endothermic character of the three adsorption mechanisms of the metals^38^.

## Conclusion

The target of this article is to investigate the metal-porphyrin complexes through the QCM measurements of the experimental adsorption isotherms of AlCl_3_, FeCl_3_ and InCl_3_ on porphyrins. Based on the adsorption capacities of the three systems, it was discovered that the AlCl_3_ was the best adsorbate that can be used for the metalloporphyrin application since the chloride ions do not have any influence on the porphyrin complexation comparing to the InCl_3_ adsorption. It was also verified that a single-layer model (ideal gas) can be used for the theoretical characterization of the AlCl_3_ adsorption indicating that there is no lateral interactions effect comparing to the FeCl_3_ system. Thus, the participation of chloride ions in the double-layers adsorption of InCl_3_ and the lateral interactions influencing the FeCl_3_ adsorption disfavors the complexation of the tested porphyrin by the two metallic ions iron(III) and indium(III).

Theoretically speaking, the steric study showed that the three complexation mechanisms took place with a multi-interaction mechanism since the number of bonded ions per site n did not exceed 1 for all the temperatures. The density of receptor sites was the highest for AlCl_3_ because the anions did not contribute at the adsorption process and the complexation mechanism of aluminum(III) took place without lateral interactions effect. The energetic analysis indicated that the interaction aluminum(III)-porphyrin can be a covalent or ionic bonds whereas the adsorption of iron and indium took place via physisorption process.

Correlating all the experimental results and the theoretical findings, one can conclude that the aluminum(III) is the appropriate material for the metal-porphyrin complex application.

## References

[1] Moro P, Donzello MP, Ercolani C, Monacelli F, Moretti G (2011). tetrakis-2,3-[5,6-di-(2-pyridyl)-pyrazino]porphyrazine, and its Cu(II) complex as sensitizers in the TiO_2_-based photo-degradation of 4-nitrophenol. J. Photochem. Photobiol. A..

[2] Sayyad MH, Saleem M, Karimov KS, Yaseen M, Ali M, Cheong KY, Mohd Noor AF (2010). Synthesis of Zn(II) 5,10,15,20-tetrakis(4′-isopropylphenyl) porphyrin and its use as a thin film sensor. Appl. Phys. A..

[3] Kadish KM, Smith KM, Guilard R (2003). The Porphyrin Handbook, Phthalocyanines: Spectroscopic and Electrochemical Characterization.

[4] Ponka P (1999). Cell Biology of heme. Am. J. Med. Sci..

[5] Lindsey JS, Woodford JN (1995). A simple method for preparing magnesium porphyrins. Inorg. Chem..

[6] Fuchs J, Weber S, Kaufmann R (2000). Genotoxic potential of porphyrin type photosensitizers with particular emphasis on 5-aminolevulinic acid: Implications for clinical photodynamic therapy. Free Radic. Biol. Med..

[7] Pereira MM, Dias LD, Calvete MJF (2018). Metalloporphyrins: bioinspired oxidation catalysts. ACS Catal..

[8] Guo M, Corona T, Ray K, Nam W (2019). Heme and nonheme high-valent iron and manganese oxo cores in biological and abiological oxidation reactions. ACS Cent. Sci..

[9] Radecka H, Grzybowska I, Radecki J, Jakubowski P, Lateran S, Orlewska C, Maes W, Dehaen W (2007). Salicylate determination in human plasma by ISEs incorporating Mn(III)-porphyrine and Zn(II)-dipyrromethene. Anal. Lett..

[10] Gorski L, Malinowska E (2005). Fluoride-selective sensors based on polyurethane membranes doped with Zr(IV)-porphyrins. Anal. Chim. Acta..

[11] Shamsipur M, Javanbakht M, Hassaninejad AR, Sharghi H, Ganjali MR, Mousavi MF (2003). Highly selective PVC-membrane electrodes based on three derivatives of (tetraphenylporphyrinato)cobalt(III) acetate for determination of trace amounts of nitrite ion. Electroanalysis.

[12] Steinle ED, Schaller U, Meyerhoff ME (1998). Response characteristics of anion-selective polymer membrane electrodes based on Gallium(III), Indium(III) and Thallium(III) porphyrins. Anal. Sci..

[13] Malinowska E, Meyerhoff ME (1995). Role of axial ligation on potentiometric response of Co(III) tetraphenylporphyrin-doped polymeric membranes to nitrite ions. Anal. Chim. Acta..

[14] Stulz E, Scott SM, Ng YF, Bond AD, Teat SJ, Darling SL, Feeder N, Sanders JKM (2003). Construction of multiporphyrin arrays using ruthenium and rhodium coordination to phosphines. Inorg. Chem..

[15] Konishi K, Makita K, Aida T, Inoue S (1988). Highly stereoselective hydrogen transfer from alcohols to carbonyl compounds catalysed by aluminium porphyrins. J. Chem. Soc. Chem. Commun..

[16] Ojima I (2000). Catalytic Asymmetric Synthesis.

[17] Bond GC (1987). Heterogeneous Catalysis: Principles and Applications.

[18] De Vos DE, Vankelecom IFJ, Jacobs PA (2000). Chiral Catalyst Immobilization and Recycling.

[19] Langmuir I (1918). The adsorption of gases on plane surfaces of glass, mica and platinium. Am. Chem. Soc..

[20] Ben Yahia M, Aouaini F, Almogait ES, Al-Ghamdi H (2019). Theoretical investigation of the chlorophyll nucleus adsorption monitored with Quartz crystal microbalance technique: new insights on physicochemical properties. J. Mol. Liq..

[21] Ben Yahia M, Tounsi M, Aouaini F, Knani S, Ben Lamine A (2017). A Statistical physics study of the interaction of [7]-helicene with alkali cations (K^+^ and Cs^+^): New insights on microscopic adsorption behavior. RSC. Adv..

[22] Bouzid M, Zhu Q, Geoff DM, Ben Lamine A (2018). New insight in adsorption of pyridine on the two modified adsorbents types MN200 and MN500 by means of grand canonical ensemble. J. Mol. Liq..

[23] Brunauer S, Emett PH, Teller E (1938). On adsorption of gases in multimolecular layers. J. Am. Chem. Soc..

[24] Buck P, Lindner E, Kutner W, Inzelt G (2004). Piezoelectric chemical sensors. Pure Appl. Chem..

[25] O’Sullivan CK, Guilbault GG (1999). Commercial quartz crystal microbalances theory and applications. Biosens. Bioelectron..

[26] Sauerbrey G (1959). Use of quartz vibration for weighing thin films of a microbalance. Z. Phys..

[27] Kôfilinger C, Drost S, Aberl F, Wolf H, Koch S, Woias P (1992). A quartz crystal biosensor for measurement in liquids. Biosens. Bioelectron..

[28] Nomura T, Okuhara M (1982). Frequency shifts of piezoelectric quartz crystals immersed in organic liquids. Anal. Chim. Acta..

[29] BenYahia M, Knani S, Hsan LBH, Nasri H, Lamine AB (2017). Statistical studies of adsorption isotherms of iron nitrate and iron chloride on a thin layer of porphyrin. J. Mol. Liq..

[30] Ben Lamine A, Bouazra Y (1997). Application of statistical thermodynamics to the olfaction mechanism. Chem. Sens..

[31] Sellaoui L, Soetaredjo EF, Ismadji S, Petriciolet BA, Belver C, Bedia J, Lamine BA, Erto A (2018). Insights on the statistical physics modeling of the adsorption of Cd^2+^ and Pb^2+^ ions on bentonite-chitosan composite in single and binary systems. Chem. Eng. J..

[32] Ben Yahia M, Hsan HBL, Knani S, Yahia MB, Nasri H, Lamine AB (2016). Modeling of adsorption isotherms of zinc nitrate on a thin layer of porphyrin. J. Mol. Liq..

[33] BenYahia M, BenYahia M, Aouaini F, Knani S, Al-Ghamdi H, Almogait ES, Lamine AB (2019). Adsorption of sodium and lithium ions onto helicenes molecules: Experiments and phenomenological modeling. J. Mol. Liq..

[34] Alyousef H, Aouaini F, Ben Yahia M (2020). New insights on physico-chemical investigation of water adsorption isotherm into seed of dates using statistical physics treatment: Pore size and energy distributions. J. Mol. Liq..

[35] Knani S, Khalifa N, BenYahia M, Aouaini F, Tounsi M (2020). Statistical physics study of the interaction of the 5, 10, 15, 20-tetrakis (4-tolylphenyl) porphyrin (H_2_TTPP) with magnesium ion: New microscopic interpretations. Arab. J. Chem..

[36] Ben Khemis I, Mechi N, Sellaoui L, Ben Lamine A (2016). Modeling of muscone enantiomers olfactory response by an adsorption process onto the mouse muscone receptor MOR215–1. J. Mol. Liq..

[37] Aouaini F, Knani S, Ben Yahia M, Ben Lamine A (2017). Statistical physics studies of multilayer adsorption isotherm in food materials and pore size distribution. Phys. A.

[38] Ayachi F, Nakbi A, Sakly A, Pinto LAA, Ben Lamine A (2019). Application of statistical physics formalism for the modeling of adsorption isotherms of water molecules on the microalgae *Spirulina platensis*. Foods. Bioprod. Process..

